# The Role of Geographical Area and Entrepreneurs' Personality

**DOI:** 10.3389/fpsyg.2021.671931

**Published:** 2021-07-23

**Authors:** Amaia Yurrebaso, Eva María Picado, Teresa Paiva

**Affiliations:** ^1^Department of Social Psychology and Antropology, University of Salamanca, Salamanca, Spain; ^2^Labour Law, Social Work and Social Services, University of Salamanca, Salamanca, Spain; ^3^Technological and Management School, CI&DEI, Guarda Polytechnic Institute, NECE–Research Center in Business Sciences, Guarda, Portugal

**Keywords:** maintaining entrepreneurial intention, entrepreneur's personality, geographical area, regional development, entrepreneurship

## Abstract

This study proposed a line of research on entrepreneurship based on the analysis of personality traits and geographical area. Its objective is to identify whether certain personality traits or sociocultural variables typical of a particular geographical area influence those who have already started an entrepreneurial activity to keep it up, in other words, to maintain their entrepreneurial intention. The research results reach a sample of 479 entrepreneurs from two Iberian Peninsula geographical areas. The analyse of the psychometric properties on the Entrepreneurial Orientation Questionnaire (EOQ) identified five dimensions of the enterprising personality. They also evidence that geographical location is a factor that contributes to the development of the entrepreneurial intentions that determine the business profile. The results show that entrepreneurs in the northern area tend to maintain their business than those in the central zone.

## Introduction

In recent years there has been growing social interest in everything that has to do with the world of entrepreneurs and the role that they can play in socio-economic life as activating agents. It has also become necessary to increase employability indices to improve the quality of life of the population, and a valid mechanism for achieving this is self-employment. The latter, in permanent transformation, stands as the great challenge that drives economic growth, which is why entrepreneurial initiative plays a significant role in the present societies (European Commission, 2012; Moriano et al., 2012; Wu et al., 2013).

Entrepreneurial behaviour, in what it implies to creating and implementing an idea or business, can be defined as planned behaviour. If the simple intention to execute conduct does not guarantee its execution, and that the intention is one of the best predictors of certain types of behaviour, we can justify the model of intentions in the field of entrepreneurship (Krueger et al., 2000; Coulibaly et al., 2018; Echeverri et al., 2018; Dencker et al., 2019; Hou et al., 2019; Tornikoski and Maalaoui, 2019). Entrepreneurship-based research highlights the contribution of intent as an individual predictor of planned behaviour. They are a coherent and robust framework that helps to understand entrepreneurial processes (Sánchez, 2005; Sánchez García, 2010; Sánchez and Yurrebaso, 2012; Coulibaly et al., 2018; Echeverri et al., 2018; Dencker et al., 2019; Hou et al., 2019; Tornikoski and Maalaoui, 2019).

Even Krueger et al. (2000) turned to the Theory of Planned Behaviour by Ajzen (1991) and Shapiro's Business Event Model (Shapiro, 1982), after confirming in their research that situational (loss of employment) and individual variables (personality traits) offer partial and inconsistent empirical results in predicting entrepreneurial behaviour. They concluded that intentions are the essential elements of entrepreneurial behaviour, over and above the situational and individual variables that indirectly influence the entrepreneur. Intention plays a mediating role between entrepreneurial action and potential external influences. The intention is the cognitive state immediately before the execution of behaviour. Attitudes influence behaviour, because of their impact on intentions, in the same way that intentions and attitudes depend on the situation. In the intention model, in addition to the variables referred to, other factors of individuals are also influential, such as their level of education, marital status, and vicarious experience (Krueger et al., 2000).

Scholten et al. (2004) also apply the Theory of Planned Behaviour to entrepreneurial behaviour. In their definition of the antecedents of intention, an “attitude for entrepreneurship” refers to the beliefs about the possible results obtained from creating a business and how these results would be evaluated. The subjective norm, which includes beliefs about the expectations of others regarding entrepreneurial behaviour and the motivations to meet these expectations, would be the image of entrepreneurship. Finally, the perceived control of this behaviour, which includes beliefs about the presence of factors that can facilitate or impede the creation of the venture, and the perceived power to control these factors, are known as control beliefs. Scholten et al. (2004) conclude that maintaining the intention to embark on an undertaking will be more likely the more favourable the attitude and the subjective norms, and the greater the control perceived by the subject (Scholten et al., 2004).

Another decisive element in the maintenance of entrepreneurial intention is found in the social aspects that directly or indirectly influence the desire, capability, and feasibility of implementing a business idea (Hurtado et al., 2007; Zahra and Nambisan, 2011; Obschonka and Stuetzer, 2017; Obschonka et al., 2019; Van de Vliert and Van Lange, 2019; GEM Global Entrepreneurship Monitor, 2020; Gieure et al., 2020). Knowledge, abilities, distinctive personality traits, and the socially perceived and transmitted image of the figure of the entrepreneur, can condition entrepreneurship and the maintenance of a company. Having patterns of reference on entrepreneurship in the immediate environment can determine one's perception of the possibilities of entrepreneurship (GEM Global Entrepreneurship Monitor, 2020) because our perception is based on the belief about it and the concrete value that we as individuals confer on it (Ajzen, 1991; Hung, 2006; Rauch and Frese, 2007; Suárez and Pedrosa, 2016; Baluku et al., 2019; Tornikoski and Maalaoui, 2019).

The social aspects that directly or indirectly influence the desire, capability, and feasibility of implementing a business idea can be decisive in maintaining the business. This is because cultural content reinforces our characteristics and certain types of behaviour while penalising others. The cultural substratum—the system underlying normative values and the models of perceiving entrepreneurial activity—plays a critical role in determining our entrepreneurial behaviour (Throsby, 2001; Zahra and Nambisan, 2011; Baluku et al., 2018, 2019).

The model differentiates between two dimensions of analysis: the intention (before the behaviour, in this case, the intention to open a business or undertaking) and the final behaviour to which the intention (start the business) is supposed to lead. This study will focus on the second dimension, given the few studies that have analysed it so far (most focus on the intention and not the realisation). To do so, it employs as a sample a group of entrepreneurs who have already opened their companies.

We understand that the entrepreneur has different personality traits, some innate and others socially learned, values, social patterns, and cultural factors that contribute to the configuration of that personality. In addition to the personality traits, we examine the potential effects of the geographical location of the company on maintaining entrepreneurial intention. Given the exposure, this paper aimed to study the role of geographical area and the possession of certain personality traits in maintaining entrepreneurial intention.

Although the Iberian Peninsula indeed shows levels of entrepreneurial activity similar to those of other countries with the same degree of economic development, the existence of solid regional inequalities in its evolution, both in the level of entrepreneurship and in the type of activity undertaken, is evident. According to the GEM report on Spain (2020), the central area of a country shows a lower rate of entrepreneurial activity. The northern region has a higher rate of entrepreneurial activity, and the average size of its companies is bigger.

The novelty of our study lies in the confirmation that the differential characteristics of a geographical area contribute to the choice of location and maintenance of a business activity, which could contribute to profiling different contents in institutional policies focused on the promotion of entrepreneurship.

This paper begins by analysing the theoretical framework of geographical area, assuming that each region has its cultural factors, to go on to that of the entrepreneurial personality. This will lead to the hypotheses formulation that supports the model proposed. After analysing the methodological characteristics of our study, we present the results obtained and the main conclusions and implications deriving from them.

## Geographical Area and Personality Traits: A Theoretical Review

Certain complementary sociodemographic factors can contribute to developing personality traits, jointly affecting the formation and maintenance of entrepreneurial intention through its effects on the antecedents of intention (Fishbein Ajzen, 2010).

### Geographical Area and Regional Culture

Many scholars assume that cultural factors are crucial for the development of economic activity in general (Beugelsdijk, 2007; Rentfrow et al., 2013) and the fostering of entrepreneurship in particular (Davidsson, 1995; Davidsson and Wiklund, 1997; Audretsch, 2007; Nunn, 2009; Kibler et al., 2014; Obschonka et al., 2015; Obschonka, 2017). Given the growing interest of different disciplines in this area, psychological research, based on Big Data, is attempting, although still with certain limitations, to use personality studies to assess the origins and possible effects of an enterprising culture, in the assumption that entrepreneurship could be motivated by local culture (Fritsch and Wyrwich, 2013; Hayton and Cacciotti, 2013; Obschonka, 2017).

A group approach assumes that the inhabitants of a town, region, or area would share and be influenced by specific cultural patterns that favour entrepreneurship. Such as entrepreneurship perception, experiences, and training in business activity, role models, attitudes, and beliefs, ways of doing things. Some scholars even claim that entrepreneurship should be understood as something social and regional (Feldman, 2001) since the processes generated in business systems motivate and regulate the entrepreneurial intention (Ute Stephan and Patgak, 2016) and even the business activity of an area (Bandura, 1997; Stam, 2015).

In a recent study, Stuetzer et al. (2017) conclude that some US regions with higher scores in business culture experience more significant economic growth and even higher employment rates.

The so-called geography of personality traits reveals the existence of differences in personality profiles at the regional level in relation to entrepreneurship, analysing those cultural factors that influence individual behaviour and how these, in turn, generate local culture (Rentfrow et al., 2013, 2015; Oishi, 2014; Jokela et al., 2015; Obschonka et al., 2015, 2019).

Given the difficulty of identifying the boundaries of each area or region and the essential components of such a cultural framework, assigning to each region its cultural characteristics could help us explain the reason for their differences regarding entrepreneurship. We are saying that some regions may be immersed in a local culture that predisposes its inhabitants to act and start up new businesses (Andersson and Koster, 2011; Rentfrow et al., 2013).

Obschonka et al. (2015) assume that personality is the basic component of entrepreneurial culture, focus their attention on relatively small spatial areas, and attempt to predict regional entrepreneurial activity by focusing on the interaction between the knowledge the population has about entrepreneurship and the culture in which it is immersed. Obschonka et al. (2015) conceive of culture as the collective programming of the mind that makes it possible to differentiate the members of one group from another and evaluate the cultural characteristics mainly by analysing the values (McClelland, 1961; Hofstede, 2001) and the personality characteristics (Schwartz and Sagiv, 1995; McCrae and Terracciano, 2005) that prevail in the area. Personality characteristics are expressed through the corresponding values and norms in the region (Hofstede and McCrae, 2004; Rentfrow et al., 2008), to the point of empirically making traits at the cultural level operative as the measure of traits at the individual level.

Obschonka et al. (2015) consider knowledge as the basis for detecting opportunities when starting new business activities. In this way, those regions with high resources in knowledge can potentially have more significant business activity. This knowledge can even be a condition for the detection of business opportunities. Culture would be a mental model or set of internal representations, variable in time because of experience and training, created by the same cognitive systems that can interpret the environment and thus have a decisive impact on decision-making. These individual mental models are formed in interactions with others in the environment, thus creating the business culture of an area when it is shared by the majority of members of that region. In this way, the regions that develop knowledge and solid business culture will be more innovative, translating into greater business activity (Obschonka et al., 2015).

In short, these studies that analyse cultural evolution based on personality traits are critical in understanding the origin and effects of regional business culture. They highlight the usefulness of approaching personality in studying economic environments (Fredin and Jogmark, 2017).

For all of the above, the following hypothesis is proposed: H1. The geographical location of an enterprise contributes to maintaining entrepreneurial intention. Specifically, the entrepreneurial intention will be carried to a greater extent in the northern area than in the central area of the Iberian Peninsula.

### Entrepreneurial Personality

The study of entrepreneurs based on personality traits claims that these individuals possess a series of personal attributes that arouse in them the intention to undertake an enterprise, differentiating them from the rest of the population (Cromie and Johns, 1983; Cromie, 2000; Montoya Cardona, 2015; Suárez and Pedrosa, 2016). Other authors (Krueger et al., 2000; Loveland et al., 2005; Vargas, 2007; Chan et al., 2015; Yurrebaso et al., 2020) add that the personality traits that individuals possess are determinants for entrepreneurial behaviour to emerge.

The literature firmly accepts that cultural, situational, and social function factors are also integral components of the entrepreneurial process (Herron and Sapienza, 1992; Van de Ven, 1993; Baluku et al., 2018, 2019; Bockorny and Youssef-Morgan, 2019). But it is also suggested that, under similar circumstances, not all people become entrepreneurs, which leads one to believe that individual characteristics can be decisive (Cromie and Johns, 1983; Hmieleski and Carr, 2008; Contreras et al., 2017). Therefore, the attributes or characteristics of the individual must be an integral part of the research as a significant element in the understanding of the process of entrepreneurship (Carland et al., 1984; Johnson and Loveman, 1995; Stewart and Roth, 2001; Collins et al., 2004; Sánchez et al., 2005; Zhao et al., 2005; Chan et al., 2015).

This leads us to form our second hypothesis: H2. Personality variables are involved in maintaining entrepreneurial intention.

Given the difficulty involved in analysing an individual personality and the fact that many of these traits, in turn, may be influenced by environmental or sociocultural factors, we identify the ones that most authors (e.g., Cromie and Johns, 1983; Koh, 1996; Covin and Slevin, 1997; Cromie, 2000; Filion, 2003; Vecchio, 2003; Collins et al., 2004; Solesvik et al., 2014; Tarapuez et al., 2018; Tovar et al., 2018; Yurrebaso et al., 2020) establish as determinants of an entrepreneurial personality: self-efficacy, risk, proactivity, locus of control, and personal initiative.

#### Self-Efficacy

Self-efficacy is understood as the conviction that one can organise and execute actions to produce desirable results that affect situations that affect their lives (Bandura, 1997), taking on significant explanatory weight in entrepreneurship. It is an attribution of personal competence and control in a particular situation. It shows the perception that an individual has about their ability to carry out a specific action. Self-efficacy is thought to affect the choice of action and the amount of effort that the individual has to make, this being the basic predictor that the subject has in the choice of entrepreneurship (Bandura, 1997). We associate high levels of self-efficacy with persistence, innovative behaviours, and the recognition of entrepreneurial opportunities.

Some individuals avoid entrepreneurial action because they do not think they have the necessary skills (e.g., Chen et al., 1998; De Noble et al., 1999; Shane et al., 2003; Vecchio, 2003; Lent and Brown, 2006; McGee et al., 2009; Ruiz Arroyo et al., 2014; Luthans and Youssef-Morgan, 2017; Pease et al., 2018).

For all of the above, we propose that: H2a. People with greater self-efficacy will be more likely to maintain entrepreneurial intentions.

#### Risk-Taking Propensity and Ambiguity Tolerance

Risk-taking propensity, together with proactivity, is one of the three dimensions of entrepreneurial orientation (Covin and Slevin, 1997). It refers to the disposition of a subject to commit to sources of opportunities even when failure is a possibility and involves the willingness of an individual to take risks. This trait is understood by Cromie and Johns (1983) as the ability of enterprising individuals to seek and undertake productive opportunities and move comfortably in an uncertain environment.

Recent studies have found that tolerance and positive attitudes toward risk predict the formation of entrepreneurial intentions (Yurrebaso et al., 2020). It has likewise been found that a risk-taking propensity is positively associated with intentions of self-employment through its influence on certain predictors of these intentions, such as self-efficacy (Stewart and Roth, 2001; Zhao et al., 2005; Baron et al., 2016; Contreras et al., 2017; Pease et al., 2018).

Cromie and Johns (1983) understand ambiguity tolerance as the ability of entrepreneurs to make decisions even when lacking certain necessary information for doing so, that is, in a situation of uncertainty. They even contend that this attribute is definitive for distinguishing between entrepreneurs and non-entrepreneurs. In short, we propose that H2b. People with a greater risk-taking propensity will be more likely to maintain entrepreneurial intentions.

#### Proactivity

The scholarly literature identifies proactivity as an important driver of entrepreneurial intention. We can define it as the ability to identify different opportunities and act on them. It involves initiative, direct actions, and perseverance until a significant change is achieved. Proactivity puts the accent on anticipation, preventing problems before they occur and having a clear orientation to action (Shapero, 1982; Stewart and Roth, 2001; Sánchez et al., 2005; Neneh, 2019).

Taken up by Covin and Slevin (1997) as a basic dimension in the so-called entrepreneurial orientation, it involves perseverance, adaptability, and a high willingness to take responsibility for failure in entrepreneurship. Proactive individuals continuously seek business opportunities, as do organisations in markets (Stevenson and Jarillo, 1990; Lumpkin and Dess, 1996; Stewart and Roth, 2001; Mustafa et al., 2016; Arco-Tirado et al., 2019; Neneh, 2019). We, therefore, posit that: H2c. People with greater proactivity will be more likely to maintain entrepreneurial intentions.

#### Locus of Control

The term “locus of control” refers to the degree to which individuals believe they control their life and the events that influence them (Rotter, 1966). It has to do with the attributions that people make about the result of their actions and can refer to factors that are outside of themselves (external factors, such as chance or others) or to factors that the individual can control (internal factors, such as skills, or personal work).

Internal locus of control has been one of the most frequently mentioned psychological traits as a predictor of entrepreneurship. Hansemark (2003) concluded that the founders of new businesses have a higher level of internal locus of control than non-founders and defends the idea that the internal locus of control can be learned and developed over time. Experiences of success will help generate a sense of control over the situation, and failures will be attributed to external factors outside of one's control (Hansemark, 2003; Korunka et al., 2003).

Consequently, our hypothesis at this point is H2d. People with a greater internal locus of control will be more likely to maintain entrepreneurial intentions. Specifically, the opposite will be true for people who have a high external locus of control.

#### Personal Initiative

Frese and Fay (2001) suggest that this concept mainly reflects the need of organisations today to have an active conception of performance. The primary characteristic of personal initiative is that it is a work behaviour that is defined as self-initiated; someone doesn't need to say what should be done, and it does not form part of the job obligations or requirements. Instead of established objectives, in this case, we are talking about self-imposed objectives. Even though the ideas and objectives in question may have already been described previously, they have never been practised in that context. Furthermore, in cases in which it is very difficult for the behaviour to be self-initiated, that is, when it does not form part of the tasks that a person usually carries out, it can perhaps be employed with sub-tasks or certain aspects of the job, which are not clearly described as part of one's tasks.

The second characteristic that defines personal initiative is proactivity, which is behaviour oriented in the long term and not only in response to demand. Thus, the person with the initiative is prepared to deal immediately with both opportunities and threats/problems.

The third characteristic is persistence or the ability to overcome barriers to achieve a set goal since it is likely that when making the necessary changes to reach a new goal, things may not go well from the beginning. The last characteristic that defines personal initiative is that it must be pro-organisational behaviour. That is, it must positively affect the short-term results of the organisation.

In the specific case of entrepreneurship, we would say that initiative is a social and dynamic process in which individuals, alone or in collaboration, identify opportunities to innovate and act by transforming ideas into practical activities within a social, cultural, or economic context.

The European Commission (2012) defines entrepreneurial initiative as the propensity to induce changes in oneself, the ability to accept and support innovation caused by external factors, to welcome change, to take responsibility for one's own actions (whether positive or negative), to finish what is started, to know in which direction it is going, to establish objectives and fulfil them, and to have the necessary motivation for success.

It is in this theoretical framework that we formulate the following hypothesis: H2f. People with greater personal initiative will be more likely to maintain entrepreneurial intentions.

Now that the main theoretical concepts of this work have been defined, and in response to the interest of delimiting the influence of certain individual and cultural variables in maintaining innovative entrepreneurial intention, our starting hypotheses can be summarised thus: (a) the personality variables described are associated with the intention to continue entrepreneurship; (b) the geographical area of an entrepreneur is associated with the intention to continue with entrepreneurship. With these premises in mind, we intend to build a parsimonious model that will allow us to predict, with the least possible error, the degree to which this entrepreneurial intention is maintained.

## Method

### Participants

For this study, we carried out an incidental rather than probabilistic sampling. Through this procedure, a sample of 479 enterprising subjects was obtained, 262 of which were males (55%), 210, females (44%) and 7 did not answer (1%). Regarding the work experience of the respondents, 148 had an experience of between 1 and 10 years (31%), 183 had more than 10 years of experience (38%), and 148 did not answer (31%).

To put together the sample, the lists of companies registered in each geographical area were taken as a reference. These lists were facilitated by the different Chambers of Commerce and Industry of each province. Once obtained, a request for collaboration with the study was sent. Those who wanted to collaborate were counted for the sample and those who were founders of a company and continued to maintain links with it.

The ages of these subjects range between 19 and 76 years, with an average of 38.68 years. As for the origin of the 479 subjects in the sample, 225 came from the northern part of the Iberian Peninsula, and 254 from the central peninsular area (hereinafter referred to as “North” and “Centre”).

### Instrument

The instrument used to carry out this research was the Entrepreneurial Intent Questionnaire-COE (*Cuestionario de Intención Emprendedora* in the original Spanish) (Sánchez García, 2010), chosen for its optimal adaptation to our study purposes since it measures traits that in the literature are considered important in entrepreneurial behaviour. The structure of the questionnaire is divided into two blocks: the first section of control variables (personal data such as age, sex, type of education, employment situation, the profession of parents ...) that could have an effect on their entrepreneurial intention, and a second block with variables related to the personal profile of an entrepreneur (internal and external locus of control, entrepreneurial self-efficacy, proactivity, risk, and initiative) measured on a Likert scale (from 0 to 5).

For this study, we also used the variables of work intention to maintain one's own company, the perception of the feasibility of the idea, the attractiveness of the idea, and the support of the environment toward the idea. With the weighting of the data collected in all of these variables, a new variable was calculated: maintenance of entrepreneurial intention.

### Data Analysis

To assess the influence of the geographical area variable on the maintenance of entrepreneurial intention, a *t*-test of the difference in means was performed. Also, to deepen the analysis, a Chi-square test was run, dividing the variable “maintenance of entrepreneurial intention” into three categories (low-medium-high). To analyse whether there were differences in the personality variables (ILC, ELC, Self-efficacy, Proactivity, Risk, and Personal Initiative), a one-way ANOVA was performed in which subjects who had maintained a high entrepreneurial intention were compared to those with a low entrepreneurial intention. Finally, a logistic regression analysis was carried out to construct a model in which the geographical area variable, as well as personality variables, come into play.

Psychometric evaluation of scales is set out in Table 1. Regarding reliability, taking as reference the Crombach Alpha, none of the values is <0.7. As for the one-dimensionality of the scales, LCE, that of LCI, Self-Efficacy, and Proactivity, seems more than an acceptable hypothesis, even though they all have at least two self-values above one. This one-dimensionality would not be so clear for the last scales. The difference between the variance that explains the first factor and the one that explains the second is not so great.

**Table 1:** Psychometric evaluation of scales.

	**Number of items on the scale**	**Reliability (Crombach Alpha)**	**KMO/Bartlett's Sphericity Test**	**Scale One-Dimensionality (CP.)**	**χ^2^**	**Confirmatory model adjustment indicators**	**Covariance between mistakes**
LCI	11	0.84	0.89/χ^2^ = 1,601; *p* < 0.00	2 self-values >1. The first explains 41% variance and the second 21%	169 (*p* < 0.00)	Cmin/df = 4, NFI = 0.9 and RMSEA = 0.08	Between the error of item 6 and item 11 and between the error of item 11 and the error of item 15
LCE	8	0.81	0.83/χ^2^ = 1,525; *p* < 0.00	2 self-values >1. The first explains 44% variance and the second 23%	35 (*p* < 0.00)	Cmin/df = 2.5, NFI = 0.98 and RMSEA = 0.06	Between the error of item 3 and item 7, between the error of item 3 and the error of item 12, between the error of item 3 and item 16, between the error of item 7 and the error of item 12, between the error of item 7 and the error of item 16 and between the error of item 12 and the error of item 16
Self-efficacy	23	0.93	0.94/χ^2^ = 5,335; *p* < 0.00	4 self-values above 1. The first self-value would explain 40% of the variance and the second self-value would explain 8%	1,090 (*p* < 0.00)	Cmin/df = 4.8, NFI = 0.9 and RMSEA = 0.09	Between the error of item 6 and item 12, between the error of item 7 and the item error 2, between the error of item 7 and item 13, between the error of item 12 and the error of item 16, and between the error of item 18 and the error of item 21
Proactivity	10	0.83	0.85/χ^2^ = 1,311; *p* < 0.00	2 self-values above 1. The first would explain 40% of the variance and the second self-value 11%	146 (*p* < 0.00)	Cmin/df = 4.3, NFI = 0.9 and RMSEA = 0.08	Between the error of item 5 and item 9
Risk propensity	11 (¬v21)	0.69	0.75/χ^2^ = 974; *p* < 0.00	3 self-values above 1. The first would explain 26% of the variance and the second self-value by 17%	144 (*p* < 0.00)	Cmin/df = 4.8, NFI = 0.84 and RMSEA = 0.08	Between item 11 and item 19, between item 12 and item 21, between 12 and 16, between 14 and 16, and between 16 and 17
Personal initiative	13	0.74	0.8/χ^2^ = 1,047; *p* < 0.00	3 self-values above 1. The first would explain 26% of the variance and the second self-value by 11%	257 (*p* < 0.00)	Cmin/df = 4.01, NFI = 0.8 and RMSEA = 0.08	Between the error of item 25 and item 35

As for The Confirmatory Factorial Analysis, all models have a significant value of χ^2^. However, the contrast of hypotheses about this value is very sensitive to certain characteristics of the sample, such as its size. Hence it has been chosen to also refer to the value of other goodness-of-fit indicators. Generally speaking, all scales have an acceptable fit to the one-dimensional model proposed for each of the scales. The value of the NFI, taking as a reference 0.09, would notify us that there are adjustment problems in the Scale of Risk Propensity and Personal Initiative. On the other hand, the value of RMSA, taking as reference 0.08, would warn us of a possible problem of adjustment in the Self-efficacy scale. These possible mismatches will be taken into account when interpreting, with more or less caution, the conclusions of this investigation.

## Results

### Geographical Area and Entrepreneurial Intention

**Figure 1:**
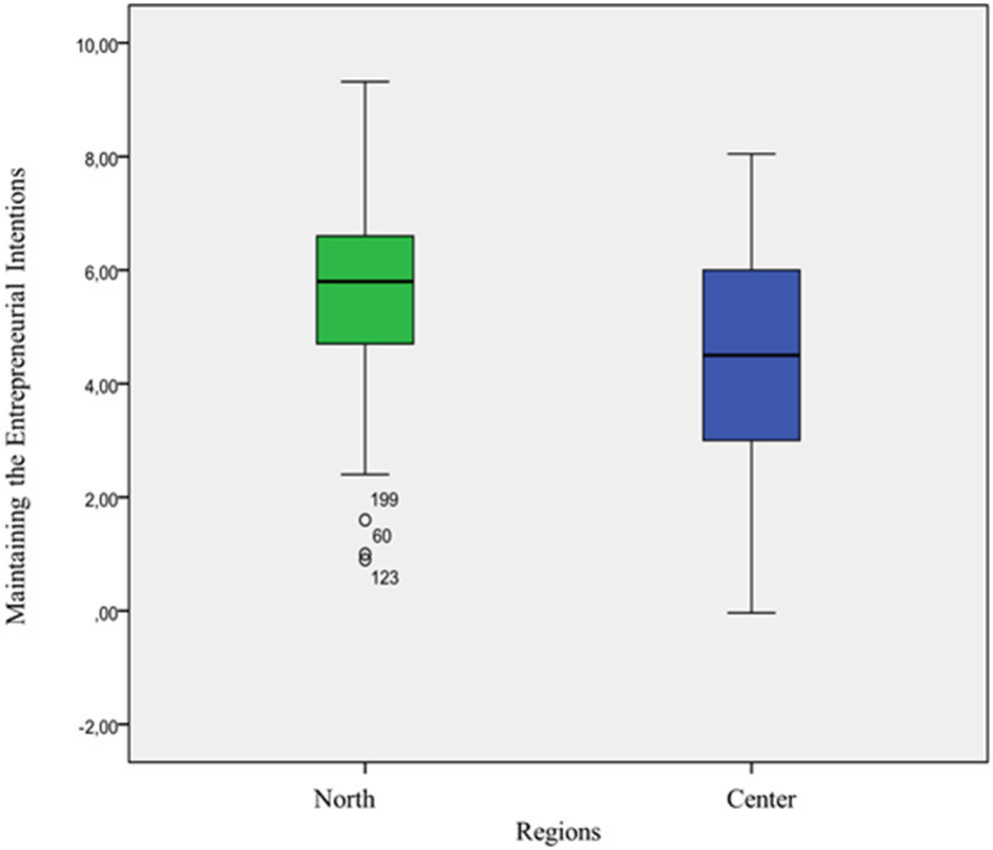
Box plot of the variable maintenance of entrepreneurial intent in the north and in the central area.

First, the relationship between the geographical area variable (North, Centre) and the maintenance of entrepreneurial intention was analysed. The average entrepreneurial intention for the North was 5.57 (5.38–5.75), and that of the Centre was 4.62 (4.14–4.38) (Figure 1).

A *t-*test for independent samples, assuming different variances, suggests significant differences between entrepreneurs in North and those in the Centre in terms of maintaining entrepreneurial intention (*t* = 7.74, *p* < 0.05).

However, to reinforce the hypothesis that there are differences in the maintenance of entrepreneurial intention between the North and the Centre subjects, we decided to categorise the maintenance of entrepreneurial intention variable to perform a χ^2^ analysis that would shed more light on this result. The new variable consisted of three categories: maintenance of low (0–3.55), medium (3.60–5.90), and high (5.91–9.32) entrepreneurial intention.

In the analysis, no expected frequency with a value lower than 5 was found, and therefore the Chi-square test results can be considered free of the problem that this circumstance could imply.

The χ^2^ value obtained differs significantly from 0 (χ^2^ = 42.3, *p* < 0.05), which means that the hypothesis of independence is rejected for the usual levels of significance. Thus, it can be affirmed that the test yields a positive result regarding the association between the maintenance of entrepreneurial intention and the geographical area of the entrepreneur. The degree of association between the two variables, taking Cramer's V as the indicator (*V* = 0.3, *p* < 0), is significant. In addition, the magnitude of Cramer's V suggests a moderate relationship between the maintenance of entrepreneurial intention variable and the variable representing the geographical area in which the entrepreneur set up their company. Furthermore, the Spearman correlation takes a value of −0.28, with a *p* < 0.01. The negative sign of the Spearman correlation tells us that subjects from the North tend to belong to a higher category in the maintenance of entrepreneurial intention variable, confirming what could already be intuited in Figure 1.

Once the value of Lamda (*L* = 0.01; *p* = 0.04) was estimated, taking the geographical area variable as dependent variable (λ area = 0.16; *p* < 0), knowledge of the classification in the maintenance of entrepreneurial intention variable makes it possible to reduce the uncertainty in the prediction of the geographical area variable in a significant way, specifically by 16%. In contrast, knowledge of the group to which a subject belongs in the geographical area variable does not make it possible to reduce the error when predicting the group to which the subject pertains in the maintenance of entrepreneurial intention variable (λ area = 0.05; *p* > 0.05). That is, the moderate relationship found between the geographical area variable and maintenance of entrepreneurial intention variable would be better reflected if we take the maintenance of entrepreneurial intention variable as an independent variable and the geographical area variable as a dependent variable, since placing the variables otherwise the difference with what would be a random distribution would not be significant.

### Personality Variables, and Maintenance of Entrepreneurial Intention

To analyse the relationship between personality variables (ILC, ELC, Self-efficacy, Proactivity, Risk Propensity, and Personal Initiative) and the categorised maintenance of entrepreneurial intention (low-medium-high) variable, one-way ANOVA was performed.

To analyse the homogeneity of variances, the Levene test was used for the ILC scale (2.2, *p* = 0.11), for the ELC scale (5.5, *p* < 0), for the Self-efficacy scale (0.92, *p* = 0.92), for the Proactivity scale (0.38, *p* = 0.38), for the Risk scale (0.43, *p* = 0.65) and for the Personal Initiative scale (0.64, *p* = 0.52). According to this test and a significance level of 0.05, the only scale that shows a variance problem would be the ELC scale.

And within the test, for the ILC variable, it was found that there were significant differences (*F* = 25.52, *p* < 0.00); for the ELC variable, it was found that there were no significant differences (*F* = 0.86, *p* = 0.43); for the Self-efficacy variable it was found that there were differences (*F* =23.02, *p* < 0); for the Proactivity variable, it was also found that there were differences (*F* = 20.62, *p* < 0.00), and the same can be said of both the Risk variable (*F* = 8.51; *p* < 0), and the personal initiative variable (*F* = 16.35, *p* < 0).

Subsequently, two more tests were run, the Tuckey test for being one of the most used and the Sheffé test for being one of the most demanding when rejecting the null hypothesis. For ILC, significant differences were found between the three groups with a level of significance of 0.05. At the same time, the means of the three groups in ILC are in the same order as the groups in terms of maintaining entrepreneurial intention. From these results, it is inferred that there is an association between ILC and maintenance of entrepreneurial intention and that this association is positive. That is, the greater the ILC, the greater the entrepreneurial intention. For ELC, no significant differences were found between any of the three groups. This means that there is no association between ELC and maintenance of entrepreneurial intention.

For Self-efficacy, significant differences were found between the three groups. The three groups are ordered in the same way in self-efficacy as in maintaining the entrepreneurial intention variable. That is, the more Self-efficacy, the more the maintenance of entrepreneurial intention.

For Proactivity, significant differences were found between the three groups. The same order is also maintained as in the maintenance of entrepreneurial intention variable, implying an association between the proactivity of an individual and the maintenance of entrepreneurial intention. Moreover, this association is positive: the greater the proactivity on the part of the entrepreneur, the greater the maintenance of the entrepreneurial intention.

For Risk propensity, significant differences were found in one of the three groups: those who maintain a low entrepreneurial intention and those with a high entrepreneurial intention. Regarding the meaning of the difference of means, it can be deduced that those who maintain a high entrepreneurial intention would be willing to assume a greater risk than those who maintain a low entrepreneurial intention.

For Personal initiative, significant differences were found between those who maintained a high entrepreneurial initiative and maintained a low entrepreneurial initiative. On the other hand, no significant differences were found between those maintaining a medium or high entrepreneurial intention. Still, there was a significant difference between those maintaining a medium and those maintaining a low entrepreneurial intention in terms of personal intention.

Finally, a predictive model was constructed using logistic regression. To do so, the subjects belonging to the intermediate group were eliminated from the sample in the maintenance of the entrepreneurial intention variable. The regression parameters were calculated using Wald's backward elimination method. In the first model, all the variables were entered. This model was significant (χ^2^ = 21.56, *p* < 0).

The second model, in which the self-efficacy variable disappeared (*W* = 0.05, *p* = 0.82) with respect to the previous one, was also significant (χ^2^ = 21.08, *p* < 0). The third model, in which the variable ELC (*W* = 0.75; *p* = 0.39) disappeared with respect to the previous one was likewise significant (χ^2^ = 20.52; *p* < 0). The fourth and final model, in which the personal initiative variable disappeared (*W* = 1.47, *p* = 0.22), was also significant (χ^2^ = 18.31; *p* < 0). The model was refined to fulfil the principle of parsimony that must prevail in all scientific methods.

In the final logistic model, only the variables ILC, Proactivity, Risk, and Geographical Area appeared, the only variables whose OR is significantly different from 1 (see Table 2). Thus, the final model can increase success by 27 (77%) points concerning what would be expected if the subjects had been randomly distributed. Taking into account the scores in the three independent variables, it correctly classifies 66.1% of the entrepreneurs who maintain a low entrepreneurial intention and 83.7% of those who maintain a high entrepreneurial intention.

**Table 2:** Logistic regression model including the Risk-taking propensity variable.

	**B**	**ET**.	**Wald**	**Sig**.	**Exp(B)**
ILC	0.14	0.04	15.33	0	1.16
PROACT	0.11	0.04	8.84	0	1.12
RISK	0.11	0.05	4.95	0.03	1.12
G.AREA(1)	1.91	0.32	35.65	0	6.73
Constant	−8.07	1.25	41.69	0	0

For each additional point on the ILC scale, the probability that a subject will pertain to the group of subjects who maintain a high entrepreneurial intention increases by 16% [Exp (B) = 1.16]. For each additional point on the scale of Proactivity, the likelihood that a subject will belong to the group of subjects who maintain a high entrepreneurial intention increases by 12% [Exp (B) = 1.12]. Finally, the probability that a subject from the North will belong to the group of entrepreneurs in which the entrepreneurial intention remains high is 6.73 [Exp (B) = 6.73] times greater than if the subject belonged to the central area.

Thus, once the parameters are estimated, the model would be configured as follows:

$$\begin{array}{l} \mathrm{Pr}\left(Y=Maintaining\;the\;entreprenurial\;intention.|x_{1},x_{2},x_{3},x_{4}\;\right)\\ \;=\frac{1}{1+\exp\left(-8,07+0.14^{*}x_{1}+0.11^{*}x_{2}+0.11^{*}x_{3}+1.91^{*}x_{4}\right)} \end{array}$$

where x1 is the score of the subject in the ILC variable; x2 is the score of the subject in the Proactive variable (PROACTV); x3 is the score of the subject in the Risk variable, and x4 is the score of the subject in the Geographical area variable (0 = Centre; = 1 North).

Since the Hosmer and Leme show test gave a significant χ^2^ value and the Wald statistic was not significant for a confidence level of 99%, a new model was calculated excluding the Risk variable. For this model, the χ^2^ improved significantly (χ^2^ = 18.31, *p* = 0.02 –> χ^2^ = 14.25, *p* = 0.08), and also the *R*^2^ of Cox and Snell only decreased by one hundredth (0.3 –> 0.29) and the *R*^2^ of Nagelkerke barely decreased by another thousandth (0.40 –> 0.39). Regarding the percentage of success in the classification, it decreased only one point (78.3% –> 77.2%). The omnibus test for both models suggests that in both, there is at least one variable associated with the maintenance of entrepreneurial intention (Model: χ^2^ = 102.35, *p* < 0). Mathematically, this new model would be defined by the estimated parameters that appear in Table 3.

**Table 3:** Final logistic regression model.

	**B**	**ET**.	**Wald**	**Sig**.	**Exp(B)**
G.AREA(1)	1.86	0.31	35.2	0.00	6.44
ICI	0.14	0.04	15.13	0.00	1.15
PROACTV	0.14	0.04	13.61	0.00	1.15
Constant	−7	1.1	40.34	0.00	0

The advantage of this last model over the previous one, which justifies its calculation and exposition, is that it makes it possible to classify entrepreneurs in the maintenance of entrepreneurial intention variable with an error very similar to the previous model. Thus, with this new model, fewer resources would be needed to achieve a very similar predictive power.

## Discussion and Conclusions

The most relevant finding of this work has to do with the fact that both the social aspects, represented by the Geographical area variable, and some of the personality variables, identified by different authors as being significantly associated with entrepreneurial intention, are also related to the maintenance of a business initiative (Cromie and Johns, 1983; Covin and Slevin, 1989; Koh, 1996; Cromie, 2000; Throsby, 2001; Filion, 2003; Vecchio, 2003; Collins et al., 2004; Sánchez García, 2010; Zahra and Nambisan, 2011; Sánchez and Yurrebaso, 2012; Montoya Cardona, 2015; Mustafa et al., 2016; Baluku et al., 2018, 2019; Arco-Tirado et al., 2019; Neneh, 2019; Yurrebaso et al., 2020).

In relation to the Geographical area variable, following the momentum generated by the so-called geographical economy, which contends that within each geographical environment, there are differences that are projected directly onto the economic structure of the place in question (Gertler and Barnes, 1999; Kautonen et al., 2015; Obschonka et al., 2015, 2019; Acs et al., 2017; Ferrando et al., 2019; Guerrero and Santamaría, 2020), it was understood as a starting hypothesis for this research that there would be differences in the maintenance of entrepreneurial intention between entrepreneurs who had started their activity in the northern area and those who had started it in the centre area.

In this regard, the results of this work show that there are differences in the maintenance of entrepreneurial intention and that these differences are favourable to entrepreneurs in the North. In turn, these results would support the need to explore the business economic framework from a molecular or regional perspective (Albertos, 2002; Tarapuez et al., 2018; Guerrero and Santamaría, 2020).

As to the origin of these differences, the causes could be several, ranging from differences in grants and subsidies—the direct effect of the role that political institutions can play—to the impact of good business education (Obschonka et al., 2015; GEM Global Entrepreneurship Monitor, 2020) in which innovative ideas are rewarded (Wu et al., 2008; Dilli and Westerhuis, 2018).

Following the line defended by several authors (e.g., Krueger et al., 2000; Chan et al., 2015; Yurrebaso et al., 2020), according to which the personality traits that characterise people are important modulators of entrepreneurial intention, in this research, we found that in the variables Internal Locus of Control, External Locus of Control, Self-efficacy, Proactivity, Risk and Personal Initiative there are differences between those entrepreneurs who maintain allow entrepreneurial intention vis-à-vis those entrepreneurs who maintain a high entrepreneurial intention. In this regard, entrepreneurs who maintained a higher intention also scored higher in these variables, except in External Locus of Control. Thus, the profile of the entrepreneur who maintains a high entrepreneurial intention would be characterised by a high Internal Locus of Control, a low External Locus of Control, and high Self-efficacy, Proactivity, Risk, and Personal Initiative as compared to those with low entrepreneurial intention as regards these same variables.

On the other hand, when taking the two types of variables (geographical area and personality traits), it was found that in the most parsimonious logistic model of dichotomous classification (high entrepreneurial intention to continue—low entrepreneurial intention to continue), the variables Internal Locus of Control, Proactivity and Geographical area stood out from the rest. Moreover, the positive aspect of this model is that, if we look at the psychometric data of these scales, in particular, we find that they have acceptable validity and reliability. Future research would be necessary to investigate whether an improvement in the design of these scales, specifically adapted to the population that has already opened a business, would yield better fitted data for the model or favour the inclusion or exclusion of some other variable.

In an adaptation of the role model theory, which states that an entrepreneurial attitude would be favoured if other entrepreneurs could be imitated, that is, by the availability of entrepreneurial role models, future research could cheque whether the model itself influences the maintenance of entrepreneurial intention and, if so, identify in which ways it does so.

The research findings help entrepreneurship promotion policies define how to assist some regions in enhancing their support policies to business creation and, therefore, try to overcome regional disparities. Regarding the regional cultural differences, the policymakers may also seek to stimulate their regional societies to become more entrepreneurial and consequently diminish economic regional disparities.

## Data Availability Statement

The raw data supporting the conclusions of this article will be made available by the authors, without undue reservation.

## Author Contributions

AY as the lead author of the article “The Role of Geographical Area and Entrepreneurs' Personality,” declare that this article is unpublished, and that EP and TP contributed in the same way to its writing.

## Conflict of Interest

The authors declare that the research was conducted in the absence of any commercial or financial relationships that could be construed as a potential conflict of interest.

## Publisher's Note

All claims expressed in this article are solely those of the authors and do not necessarily represent those of their affiliated organizations, or those of the publisher, the editors and the reviewers. Any product that may be evaluated in this article, or claim that may be made by its manufacturer, is not guaranteed or endorsed by the publisher.

## References

[B1] Acs Z. Stam E. Audretsch D. B. O'Connor A. (2017). The lineages of the entrepreneurial ecosystem approach. Small Bus Econ. 49, 1–10. 10.1007/s11187-017-9864-8

[B2] Ajzen I. (1991). The theory of planned behavior, organizational behavior and human decision processes. Organiz. Behav. Human Decision Process. 50, 179–211. 10.1016/0749-5978(91)90020-T

[B3] Albertos J. M. (2002). Cultura, innovación y desarrollo local. Boletín de la AGE 34, 229–244. Available online at: https://dialnet.unirioja.es/descarga/articulo/660078.pdf

[B4] Andersson M. Koster S. (2011). Sources of persistence in regional start-up rates—evidence from Sweden. J. Econ. Geogr. 11, 179–201. 10.1093/jeg/lbp069

[B5] Arco-Tirado J. L. Bojica A. Fernández-Martín F. Hoyle R. H. (2019). Grit como predictor del emprendimiento y el autoempleo en España. Parte Delantera. Psychol. 10:389. 10.3389/fpsyg.2019.00389

[B6] Audretsch D. (2007). Entrepreneurship capital and economic growth. Oxf. Rev. Econ. Pol. 23, 63–78. 10.1093/oxrep/grm001

[B7] Baluku M. M. Kikooma J. F. Bantu E. Onderi P. Otto K. (2019). Impacto de las orientaciones culturales personales y la inteligencia cultural en el éxito subjetivo en el trabajo por cuenta propia en sociedades multiétnicas. J. Glob. Entrep. Res. 9, 8–30. 10.1186/s40497-018-0144-0

[B8] Baluku M. M. Kikooma J. F. Bantu E. Otto K. (2018). Capital psicológico y resultados empresariales: el papel moderador de las competencias sociales de los propietarios de microempresas en África Oriental. J. Glob. Entrep. Res. 8:26. 10.1186/s40497-018-0113-7

[B9] Bandura A. (1997). Self –Efficacy: The Exercises of Control. New York, NY: Freeman.

[B10] Baron R. A. Franklin R. J. Hmieleski K. M. (2016). Por qué los empresarios a menudo experimentan niveles de estrés bajos, no altos: los efectos conjuntos de la selección y el capital psicológico. J. Manag. 42, 742–768. 10.1177/0149206313495411

[B11] Beugelsdijk S. (2007). Entrepreneurial culture, regional innovativeness and economic growth. J. Evol. Econ. 17, 187–210. 10.1007/s00191-006-0048-y

[B12] Bockorny K. Youssef-Morgan C. M. (2019). Coraje, capital psicológico y satisfacción con la vida de los emprendedores. Parte Delantera. Psychol. 10:789. 10.3389/fpsyg.2019.00789 PMC6461011 31024410

[B13] Carland J. W. Boulton W. R. Carland J. A. C. (1984). Differentiating intrapreneurs from small business owners: a conceptualisation. Acad. Manage. Rev. 9, 354–359. 10.5465/amr.1984.4277721

[B14] Chan K. Y. Uy M. A. Chernyshenko O. S. Ho M. H. R. Sam Y. L. (2015). Personalidad y motivaciones emprendedoras, profesionales y de liderazgo. Pers. Individ. Diferir de. 77, 161–166. 10.1016/j.paid.2014.12.063

[B15] Chen C. Greene P. Crick A. (1998). Does entrepreneurial self-efficacy distinguish entrepreneurs from managers? J. Bus Venturing 13, 295–316 10.1016/S0883-9026(97)00029-3

[B16] Collins C. J. Hanges P. J. Locke E. A. (2004). The relationship of achievement motivation to entrepreneurial behaviour: a meta-analysis. Human Perform. 17, 95–117. 10.1207/S15327043HUP1701_5

[B17] Contreras F. de Dreu I. Espinosa J. C. (2017). Examinando la relación entre capital psicológico e intención empresarial: un estudio exploratorio. Asian Soc. Sci. 13, 80–88. 10.5539/ass.v13n3p80

[B18] Coulibaly S. K. Erbao C. Metuge Mekongcho T. (2018). Economic globalisation, entrepreneurship, and development. Tech. Forecasting Soc. Change 127, 271–280. 10.1016/j.techfore.2017.09.028

[B19] Covin J. G. Slevin D. P. (1989). Strategic management of small firms in hostile and benign environments. Strateg. Manage. J. 10, 75–87.

[B20] Covin J. G. Slevin D. P. (1997). High growth transitions: theoretical perspectives and suggested directions, in Entrepreneurship 2000, eds Sexton D. L. Smilor R. (Chicago: Upstart), 99–126.

[B21] Cromie S. (2000). Assessing entrepreneurial inclinations: some approaches and empirical evidence. Eur. J. Work Organiz. Psychol. 9, 7–30. 10.1080/135943200398030

[B22] Cromie S. Johns S. (1983). Irish entrepreneurs: some personal characteristics. J. Occupat. Behav. 4, 317–324.

[B23] Davidsson P. (1995). Culture, structure and regional levels of entrepreneurship. Entrep. Reg. Dev. 7 41–62. 10.1080/08985629500000003

[B24] Davidsson P. Wiklund H. (1997). Values, beliefs and regional variations in new firm formation rates. J. Econ. Psychol. 18, 179–199.

[B25] De Noble A. F. De Noble A. F. Jung J. Ehrlich S. B. (1999). Entrepreneurial self-Efficacy: the development of a measure and its relationship to entrepreneurial action, in Frontiers of Entrepreneurship Research-1999, eds Reynols P. D. Bygrave W. D. Manigart S. Manson C. M. Meyer G. D. Sapienza H. J. Shaver y K. S. (Wellesley MA: Babson College), 73–87.

[B26] Dencker J. Bacq S. C. Gruber M. Haas M. (2019). Reconceptualising necessity entrepreneurship: a contextualised framework of entrepreneurial processes under the condition of basic needs. Acad. Manage. Rev. amr.2017.0471. 10.5465/amr.2017.0471

[B27] Dilli S. Westerhuis G. (2018). How institutions and gender differences in education shape entrepreneurial activity: a cross-national perspective. Small Bus Econ. 51, 371–392. 10.1007/s11187-018-0004-x

[B28] Echeverri L. Valencia A. Benjumea M. Barrera A. (2018). Factores que inciden en la intención emprendedora del estudiantado universitario: un análisis cualitativo. Revista Electr. Educare 22:1. 10.15359/ree.22-2.10

[B29] European Commission (2012). Effects and Impact of Entrepreneurship Programmes in Higher Education. Brussels: European Commission.

[B30] Feldman M. P. (2001). The entrepreneurial event revisited: firm formation in a regional context. Industr. Corporate Change 10, 861–891. 10.1093/icc/10.4.861

[B31] Ferrando S. Velilla J. Ortega R. (2019). Intergenerational transmission of entrepreneurial activity in spanish families. J. Family Econ. 40, 390–407. 10.1007/s10834-019-09613-7

[B32] Filion L. J. (2003). Emprendedores y propietarios dirigentes de pequeñas y medianas empresas (PME). Revista de Administr. de Empr. 34,5–28. Available online at: https://rayo.xoc.uam.mx/index.php/Rayo/article/view/301

[B33] Fishbein M. Ajzen I. (2010) Revisión de predecir y cambiar el comportamiento: el enfoque de acción razonada. New York, NY: Psychology Press; Taylor & Francis Group, 518.

[B34] Fredin S. Jogmark M. (2017). Local culture as a context for entrepreneurial activities. Eur. Plann. Stud. 25, 1556–1574. 10.1080/09654313.2017.1306028

[B35] Frese M. Fay D. (2001). Personal initiative (PI): an active performance concept for work in the 21st Century. Res. Organisat. Behav. 23, 133–187. 10.1016/S0191-3085(01)23005-6

[B36] Fritsch M. Wyrwich M. (2013). The long persistence of regional levels of entrepreneurship:Germany,1925–2005. Reg. Stud. 48, 955–973. 10.1080/00343404.2013.816414

[B37] GEM Global Entrepreneurship Monitor (2020). Available online at: https://www.gem-spain.com/wp-content/uploads/2020/06/Informe-GEM-Espa%C3%B1a-2019_20.pdf

[B38] Gertler M. S. Barnes T. J. (Eds.) (1999). The New Industrial Geography: Regions, Regulations and Institutions. Routledge.

[B39] Gieure C. Benavides M. Roig S. (2020). The entrepreneurial process: the link between intentions and behavior. J. Bus Res. 112, 541–548. 10.1016/j.jbusres.2019.11.088

[B40] Guerrero M. Santamaría C. A. (2020). Ecosistema y actividad emprendedora en México: un análisis exploratorio. Perfiles Latino Americanos 28, 227–251. 10.18504/pl2855-009-2020

[B41] Hansemark O. C. (2003). Need for achievement, locus of control and the prediction of business start-ups: a longitudinal study. J. Econ. Psychol. 24, 301–319. 10.1016/S0167-4870(02)00188-5

[B42] Hayton J. Cacciotti G. (2013). Is there an entrepreneurial culture? A reviewofempiricalresearch. Entrep. Reg. Dev. 25, 9–10. 10.1080/08985626.2013.862962

[B43] Herron L. Sapienza H. J. (1992). The entrepreneur and the initiation of new venture launch activities. Entrepreneurship 17, 49–55. 10.1177/104225879201700106

[B44] Hmieleski K. M. Carr J. C. (2008). La relación entre el capital psicológico de los emprendedores y el desempeño de nuevas empresas. Front. Invest. Sobre Emprend. 28:1–15. Available online at: https://papers.ssrn.com/sol3/papers.cfm?abstract_id=1346023

[B45] Hofstede G. (2001). Culture's Consequences: Comparing Values, Behaviors, Institutions, and Organisations Across Nations.2 ns Edn. Thousand Oaks: Sage Publications.

[B46] Hofstede G. McCrae R. R. (2004). Personality and culture revisited: linking traits and dimensions of culture. Cross-Cult. Res. 38 52–88. 10.1177/1069397103259443

[B47] Hou F. Su Y. Lu M. Qi M. (2019). Modelo de intención empresarial de estudiantes universitarios en el delta del río Pearl de China. Parte Delantera. Psychol. 10:916. 10.3389/fpsyg.2019.00916

[B48] Hung H. (2006). Formation and survival of new ventures: a path from interpersonal to interorganizational networks. Int. Small Bus J. 24, 359–378. 10.1177/0266242606065508

[B49] Hurtado N. E. Cordón E. Senise M. E. (2007). Efectos de la cultura nacional en la relación entre orientación emprendedora y el resultado de la innovación de producto: el caso del sector farmacéutico. Cuadernos económicos de ICE 73, 135–150. Available online at: https://dialnet.unirioja.es/servlet/articulo?codigo=2339647

[B50] Johnson S. Loveman G. (1995). Starting over in Eastern Europe: Entrepreneurships and economic renewal. Boston: Harvard Business Scholl Press.

[B51] Jokela M. Bleidorn W. Lamb M. Gosling S. Rentfrow P. (2015). Geographically varying associations between personality and life satisfaction in the London metropolitan area. Proc. Natl. Acad. Sci. U.S.A. 112, 725–730. 10.1073/pnas.1415800112 25583480 PMC4311843

[B52] Kautonen T. Gelderen M. V. Fink M. (2015). Robustez de la teoría del comportamiento planificado en la predicciín de intenciones y acciones emprendedoras. Entrep. Teoría Pract. 39, 655–674. 10.1111/etap.12056

[B53] Kibler E. Kautonen T. Fink M. (2014). Regional social legitimacy of entrepreneurship: implications for entrepreneurial intention and start-up behavior. Reg. Stud. 48, 995–1015. 10.1080/00343404.2013.851373

[B54] Koh H. (1996). Testing hypothesis of entrepreneurial characteristics: a study of Hong Kong MBA students. J. Manag. Psychol. 11, 12–25. 10.1108/02683949610113566

[B55] Korunka C. Frank H. Lueger M. Mugler J. (2003). The entrepreneurial personality in the context of resources, environment, and the startup process-A configurational approach. Entrepreneur Theory Pract. 28, 23–42. 10.1111/1540-8520.00030

[B56] Krueger N. F. Reilly M. D. Carsrud A. L. (2000). Competing models of entrepreneurial intentions. J. Bus. Venturing 15, 411–432. 10.1016/S0883-9026(98)00033-0

[B57] Lent R. W. Brown S. D. (2006). Integrating person and situation perspectives on work satisfaction: a social-cognitive view. J. Vocational Behav. 69, 236–247. 10.1016/j.jvb.2006.02.006

[B58] Loveland J. M. Gibson L. W. Lounsbury J. W. Huffstetler B. C. (2005). Rasgos de personalidad amplios y estrechos en relación con el desempeño laboral de los consejeros de campamento. Child Youth Care Forum. 34, 241–255. 10.1007/s10566-005-3471-6

[B59] Lumpkin G. T. Dess G. G. (1996). Clarifying the entrepreneurial orientation construct and linking it to performance. Acad. Manage. Rev. 21, 135–172. 10.5465/amr.1996.9602161568

[B60] Luthans F. Youssef-Morgan C. M. (2017). Psychological capital: an evidence-based positive approach. Annu. Rev. Org. Psychol. Org. Behav. 4, 339–366. 10.1146/annurev-orgpsych-032516-113324

[B61] McClelland D. C. (1961). The Achieving Society. Princeton, NJ: D. Van Norstrand Company. Inc. (196l). 10.1037/14359-000

[B62] McCrae R. Terracciano A. (2005). 79 members of the personality profiles of cultures, project personality profiles of cultures. Aggregate personality traits. J. Pers. Soc. Psychol. 89, 407–425. 10.1037/0022-3514.89.3.407 16248722

[B63] McGee J. E. Peterson M. Mueller S. L. Sequeira J. M. (2009). Entrepreneurial self-efficacy: refining the measure. Entrepreneur. Theory Pract. 33, 965–988. 10.1111/j.1540-6520.2009.00304.x

[B64] Montoya Cardona R. A. (2015). Orientación Emprendedora Y Capacidades Para el Emprendimiento Corporativo en las Pyme: de la intención a la acción. Universidad Eafit.

[B65] Moriano J. A. Gorgievski M. Laguna M. Stephan U. Zarafshani K. (2012). Across cultural approach to understanding entrepreneurial intention. J. Career Dev. 39, 162–185. 10.1177/0894845310384481

[B66] Mustafa M. J. Hernandez E. Mahon C. Chee L. K. (2016). Intenciones emprendedoras de estudiantes universitarios en una economía emergente. J. Entrep. Emerg. Econ. 8, 162–179. 10.3926/ic.730

[B67] Neneh B. N. (2019). Del estado de alerta emprendedor al comportamiento emprendedor: el papel del rasgo de competitividad y personalidad proactiva. Pers. Individ. Diferir de. 138, 273–279. 10.1016/j.paid.2018.10.020

[B68] Nunn N. (2009). The importance of history for economic development. Annu. Rev. Econ. 1 65–92. 10.1146/annurev.economics.050708.143336

[B69] Obschonka M. (2017). The quest for the entrepreneurial culture: Psychological Big Data in entrepreneurship research. Curr. Opin. Behav. Sci. 18, 69–74. 10.1016/j.cobeha.2017.07.014

[B70] Obschonka M. Moeller J. Goethner M. (2019). Pasión y personalidad emprendedora: el caso del emprendimiento académico. Parte delantera. Psychol. 9:2697. 10.3389/fpsyg.2018.02697

[B71] Obschonka M. Stuetzer M. (2017). Integrating psychological approaches to entrepreneurship: the Entrepreneurial Personality System (EPS). Small Bus. Econ. 49, 203–231. 10.1007/s11187-016-9821-y

[B72] Obschonka M. Stuetzer M. Gosling S. D. Rentfrow P. J. Lamb M. E. Potter J. (2015). Entrepreneurial regions: do macro-psychological cultural characteristics of regions help solve the “Knowledge Paradox” of economics? PLoS ONE 10:e0129332. 10.1371/journal.pone.0129332 26098674 PMC4476658

[B73] Oishi S. (2014). Socioecological psychology. Annual Rev. Psychol. 65, 581–609. 10.1146/annurev-psych-030413-152156 23987114

[B74] Pease P. Cunningham J. Cook C. (2018). Capital Psicológico Empresarial: La Estructura De Factores. Disponible en. Available online at: http://nrl.northumbria.ac.uk/37787/

[B75] Rauch A. Frese M. (2007). ¿Nacido para ser emprendedor? Revisando el enfoque de la personalidad para el emprendimiento, en La psicología del emprendimiento, in Las Fronteras Organizativas. eds Baum J. R. Frese M. Baron R. (Nueva Jersey, NJ: Psychology Press), 41–65.

[B76] Rentfrow P. Gosling S. Jokela M. Stillwell D. Kosinski M. Potter J. (2013). Three psychological regions of the United States and their political, economic, social and health correlates. J. Pers. Soc. Psychol. 105 996–1012. 10.1037/a0034434 24128185

[B77] Rentfrow P. Gosling S. Potter J. (2008). A theory of the emergence, persistence, and expression of geographic variation in psychological characteristics. Perspect. Psychol. Sci. 3, 339–369. 10.1111/j.1745-6924.2008.00084.x 26158954

[B78] Rentfrow P. Jokela M. Lamb M. (2015). Regional personality differences in great britain. PLoS ONE 10:e0122245. 10.1371/journal.pone.0122245 25803819 PMC4372610

[B79] Rotter J. B. (1966). Generalised expectancies for internal versus external control of reinforcement. Psychol. Monogr. 80, 1–28 10.1037/h0092976 5340840

[B80] Ruiz Arroyo M. Fuentes Fuentes M. Ruiz Jiménez J. (2014). Análisis del emprendedor potencial: Integración de cognitivos y relacionales. AJOICA 12, 37–51.

[B81] Sánchez García J. C. (2010). Evaluación de la personalidad emprendedora: validez factorial del cuestionario de orientación emprendedora (COE). Rev. Latinoamer. de Psicol. 42, 41–52. Available online at: http://www.scielo.org.co/pdf/rlps/v42n1/v42n1a04.pdf

[B82] Sánchez J. (2005). Intenciones Emprendedoras en los Universitarios Castellanoleoneses. Salamanca: Cátedra de Emprendedores. Available online at: https://dialnet.unirioja.es/descarga/articulo/5610764.pdf

[B83] Sánchez J. C. Lanero A. Yurrebaso A. (2005). Variables determinantes de la creación de empresas en el contexto universitario. Revista de Psicol. Soc. Aplicada 15, 37–60.

[B84] Sánchez J. C. Yurrebaso A. (2012). La Motivación y la intención emprendedora. Int. J. Dev. Educ. Psychol. 1, 521–531.

[B85] Scholten V. Kemp R. Omta S. W. F. (2004). Entrepreneurship for life: entrepreneurial intention among academics in the Life Sciences, in Paper prepared for 2nd ESU Conference, Enschede.

[B86] Schwartz S. Sagiv L. (1995). Identifying culture specifics in the content and structure of values. J. Cross Cult. Psychol. 26 92–116. 10.1177/0022022195261007

[B87] Shane S. Locke E. A. Collins C. J. (2003). Entrepreneurial motivation. Hum. Resour. Manage. Rev. 13, 257–279. 10.1016/S1053-4822(03)00017-2

[B88] Shapero A. (1982). Social dimensions of entrepreneurship, in The Encyclopedia of Entrepreneurship, eds Kent C. Sexton D. Vesper K. 72–90.

[B89] Shapiro A. (1982). Social dimensions of entrepreneurship, in The Encyclopedia of Entrepreneurship, eds Kent C. Sexton D. Vesper K. 72–90.

[B90] Solesvik M. Westhead P. Matlay H. (2014). Factores culturales e intención emprendedora: el papel de la educación emprendedora. Educ. Entrenar. 56, 680–696. 10.1108/ET-07-2014-0075

[B91] Stam E. (2015). Entrepreneurial ecosystem sand regional policy: a sympathetic critique. Eur. Plan Stud. 23, 1759–1769. 10.1080/09654313.2015.1061484

[B92] Stevenson H. H. Jarillo J. C. (1990). A paradigm of entrepreneurship: Entrepreneurial management. Strategic Manage. J. 11:1727.

[B93] Stewart W. Roth P. (2001). Risk taking propensity differences between entrepreneurs and, managers: A meta-analytic review. J. Appl. Psychol. 86, 145–153. 10.1037/0021-9010.86.1.145 11302226

[B94] Stuetzer M. Audretsch D. Obschonka M. Gosling S. Rentfrow P. Potter J. (2017). Entrepreneurial culture, Knowledge spillovers, and the growth of regions. Reg. Stud. 52, 608–618. 10.1080/00343404.2017.1294251

[B95] Suárez J. Pedrosa I. (2016). The assessment of entrepreneurial personality: the current situation and future directions. Papeles Del Psicólogo 37, 62–68. Available online at: http://www.scielo.org.co/pdf/rlps/v42n1/v42n1a04.pdf

[B96] Tarapuez E. García M. D. Castellano N. (2018). Aspectos socioeconómicos e intención emprendedora en estudiantes universitarios del Quindío (Colombia). Innovar 28, 123–135. 10.15446/innovar.v28n67.68618

[B97] Throsby D. (2001). Economía y Cultura. Madrid: Cambridge University Press.

[B98] Tornikoski E. Maalaoui A. (2019). Reflexiones críticas: la teoría del comportamiento planificado: una entrevista con Icek Ajzen con implicaciones para la investigación del espíritu empresarial. En t. Pequeño autobús. J. Res. Entrep. 37, 536–550. 10.1177/0266242619829681

[B99] Tovar L. Balanta S. Ordoñez J. Serna W. (2018). Factores asociados al emprendimiento por oportunidad de los colombianos restornados del exterior. Migraciones 45, 119–142. 10.14422/mig.i45.y2018.005

[B100] Ute Stephan U. Patgak S. (2016). Beyond cultural values? Cultural leadership ideals and entrepreneurship. J. Bus. Venturing. 31, 505–523. 10.1016/j.jbusvent.2016.07.003

[B101] Van de Ven A. H. (1993). The development of an infrastructure for entrepreneurship. J. Bus. Venturing 8, 211–230. 10.1016/0883-9026(93)90028-4

[B102] Van de Vliert E. Van Lange P. A. M. (2019). Psicología latitudinal: una perspectiva ecológica sobre la creatividad, la agresión, la felicidad y más allá. Perspect. Psychol. Sci. 14, 860–884. 10.1177/1745691619858067 31433723

[B103] Vargas G. (2007). Influencia de la motivacion de logro, actitud emprendedora, y autoeficacia emprendedora, sobre la intención emprendedora en los estudiantes del área de ciencias empresariales de la Universidad Nacional San Antonio Abad Del Cusco. Universidad Nacional Mayor de San Marcos. Retrieved from: http://www.cybertesis.edu.pe/sisbib/2007/leiva_uj/pdf/leiva_uj.pdf

[B104] Vecchio R. P. (2003). Entrepreneurship and leadership: common trends and common threads. Human Resource Manage. Rev. 13, 303–327. 10.1016/S1053-4822(03)00019-6

[B105] Wu S. Sizong Wu S. Lingfei (2008). The impact of higher education on entrepreneurial intentions of university students in China. J. Small Bussiness Entrreprise Dev. 15, 752–774. 10.1108/14626000810917843

[B106] Wu Y. C. J. Kuo T. Shen J. P. (2013). Exploring social entrepreneurship education from a web-based pedagogical perspective. Comput. Human Behav. 29 329–334. 10.1016/j.chb.2012.08.012

[B107] Yurrebaso A. Rodríguez-Parets C. Jáñez González A. Picado- Valverde E. Guzmán- Ordaz R. Pérez- Iglesias J. L. (2020). Personalidad emprendedora y género. Cuaderno de Relaciones Laborales 1, 85–103. 10.5209/crla.68869

[B108] Zahra S. A. Nambisan S. (2011). Entrepreneurship in global innovation ecosystems. Acad. Market. Sci. Rev. 1, 4–17. 10.1007/s13162-011-0004-3

[B109] Zhao H. Seibert S. E. Hills G. E. (2005). The mediating role of self-efficacy development of entrepreneurial intentions. J. Appl. Psychol. 90, 1265–1272. 10.1037/0021-9010.90.6.1265 16316279

